# A new hybodontiform shark (*Strophodus* Agassiz, 1838) from the Upper Jurassic of Switzerland

**DOI:** 10.1186/s13358-025-00376-3

**Published:** 2025-07-09

**Authors:** Jorge D. Carrillo-Briceño, Iwan Stössel, René Kindlimann, Christian Klug

**Affiliations:** 1 Department of Paleontology, University of Zurich, Karl-Schmid-Strasse 4, 8006 Zurich, Switzerland; 2 Department of Earth and Planetary Science, ETH Zurich, Sonneggstrasse 5, 8092 Zurich, Switzerland; 3 Haimuseum und Sammlung R. Kindlimann, Zürichstraße 58, 8607 Aathal-Seegräben, Switzerland

**Keywords:** Mesozoic, Tethys Sea, Chondrichthyes, Durophagous, Swiss Prealps, Palaeobiogeography

## Abstract

The hybodontiform shark-like *Strophodus* was a large durophagous predator with highly specialized crushing-type dentition that mainly inhabited Mesozoic marine environments for more than 130 million years, with a fossil record spanning from the Middle Triassic to the Lower Cretaceous. *Strophodus* was a geographically widespread taxon with 13 species reported from Africa, Asia, Europe, India and South America. Here, we describe a new species of *Strophodus*, which we name *Strophodus timoluebkei* sp. nov. based on three teeth from the same individual in semi-articulated position. The holotype was collected in the Prealpine Sulzfluh Limestone Formation (Middle Oxfordian to Late Tithonian), Central Switzerland. *Strophodus timoluebkei* sp. nov. currently is the only vertebrate species reported from this geological unit, and its presence suggests that this durophagous shark likely played an important role as predator of the invertebrate fauna in this ancient Tethyan tropical coastal ecosystem. The new discovery sheds additional light onto the hybodontiform paleodiversity during the Upper Jurassic.

## Introduction

The genus *Strophodus* was erected by Agassiz (1838) to include distinctive hybodontiform shark-like chondrichthyans crushing-type teeth with a high degree of crown heterodonty from the Jurassic of Europe. The discovery of isolated fin spines assigned as “diagnostic” to the genus *Asteracanthus* Agassiz, 1837, in association with isolated teeth of *Strophodus* from the Jurassic of England, led Woodward (1888, 1889) to consider the latter genus as a junior synonym of *Asteracanthus*; this taxonomic scheme remained unquestioned for more than one century. Recently, Stumpf et al. (2021) proposed to reestablish *Strophodus* from synonymy with *Asteracanthus*, considering both as distinct and thus valid genera. The presence of morphological characters in the teeth and the spine of the dorsal fins allows differentiation between *Asteracanthus* and *Strophodus* (Stumpf et al., 2023). This is supported by the discovery of a well-preserved and articulated skeleton of *Asteracanthus* from the Upper Jurassic of Germany characterized by the combination of tuberculate dorsal fin spines and multicuspid teeth (with resemblance to *Hybodus* Agassiz, 1837 and *Egertonodus* Maisey, 1987); its teeth markedly differ from the durophagous crushing-type teeth characteristic of *Strophodus* (Stumpf et al., 2021, 2023).

*Strophodus* has an extensive fossil record (mainly dominated by teeth) of more than 130 million years from the Middle Triassic (Rieppel, 1981) to the Early Cretaceous, with reports from Africa, Asia, Europe, and South America (Stumpf et al., 2023, Table 1). Thirteen species are considered valid (Bhosale et al., 2024). The fossil record of *Strophodus* is restricted mainly to isolated teeth and a few partial articulated dentitions, so the morphological characteristics of the body shape and size are unknown. Woodward (1888) reported Meckel's cartilage elements of *Strophodus* with a length of up to about 30 cm, which suggests that this shark had powerful jaws with large muscle attachment sites, as also exemplified in *S*. *smithwoodwardi* (Peyer, 1946) from Switzerland and *Strophodus* sp. described by Pfeil (2011) from the Solnhofen Archipelago. The specimen reported by Woodward (1888) led to infer a maximum body length for *Strophodus* of between two and three meters, being undoubtedly one of the larger hybodontiform shark-like chondrichthyans that have ever existed (Stumpf et al., 2023). Teeth displaying extensive wear patterns provide additional evidence for a durophagous diet (e.g., Cappetta, 2012; Rigal & Cuny, 2016; Stumpf et al., 2023).

**Fig. 1:**
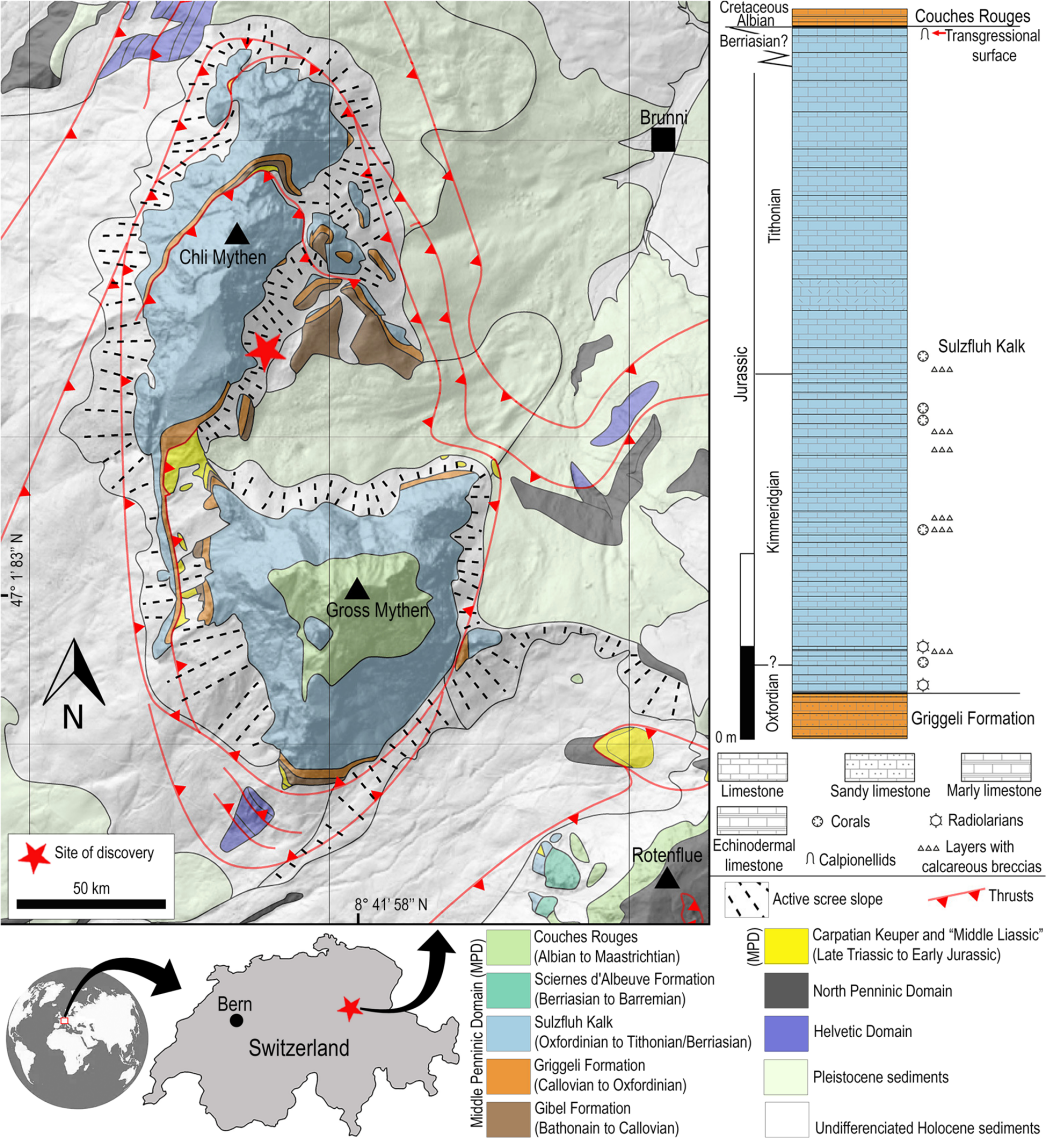
Geographical location, geological map and stratigraphic column of the Sulzfluh Limestone Fm.

Here, we describe a new species of *Strophodus* based on three complete isolated teeth from the Prealpine Sulzfluh Limestone Formation (Fm.) (Middle Oxfordian to Late Tithonian/Berriasian), Central Switzerland (Fig. 1). The new species sheds additional light onto the hybodontiform paleodiversity from the Western Tethys region during the Upper Jurassic.

## Materials and methods

### Fossil material in this study

**Fig. 2:**
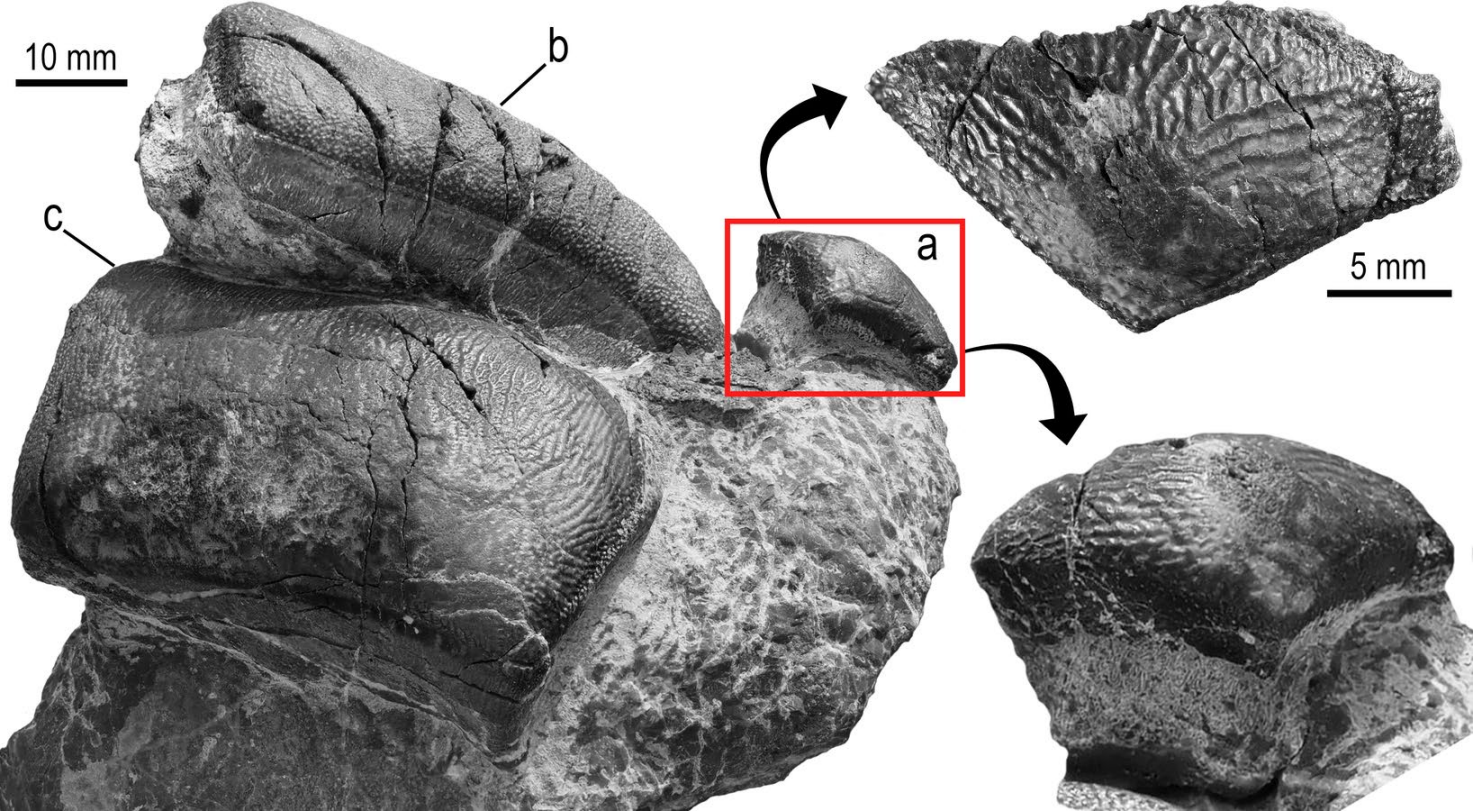
*Strophodus timoluebkei* sp. nov. and details of the anterolateral tooth PIMUZ A/I 5724-a

**Fig. 3:**
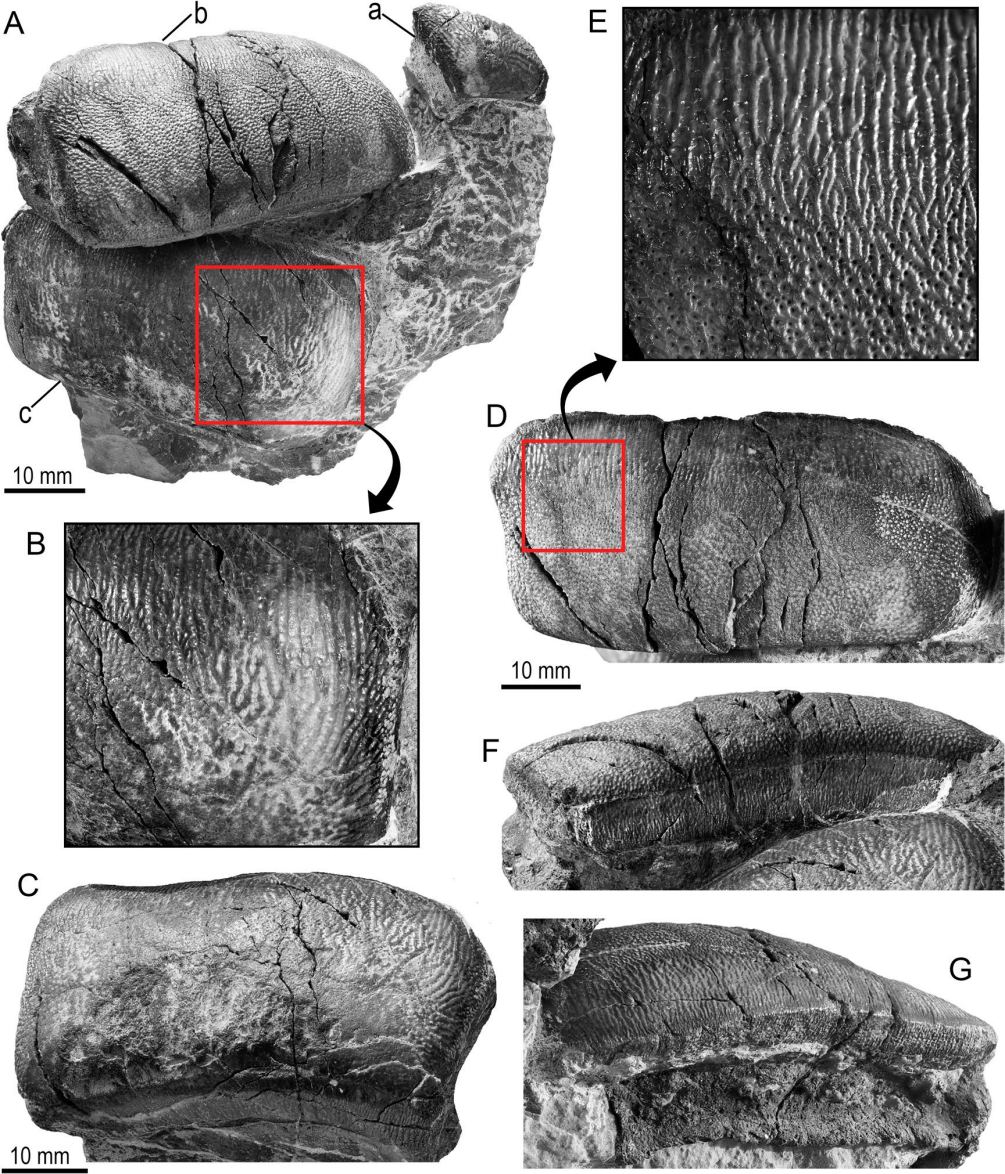
*Strophodus timoluebkei* sp. nov. and details of the lateral teeth PIMUZ A/I 5724-c (**B**, **C**) and PIMUZ A/I 5724-b (**D**–**G**). View: labial (**F**), lingual (**G**), occlusal (**A**–**E**)

**Fig. 4:**
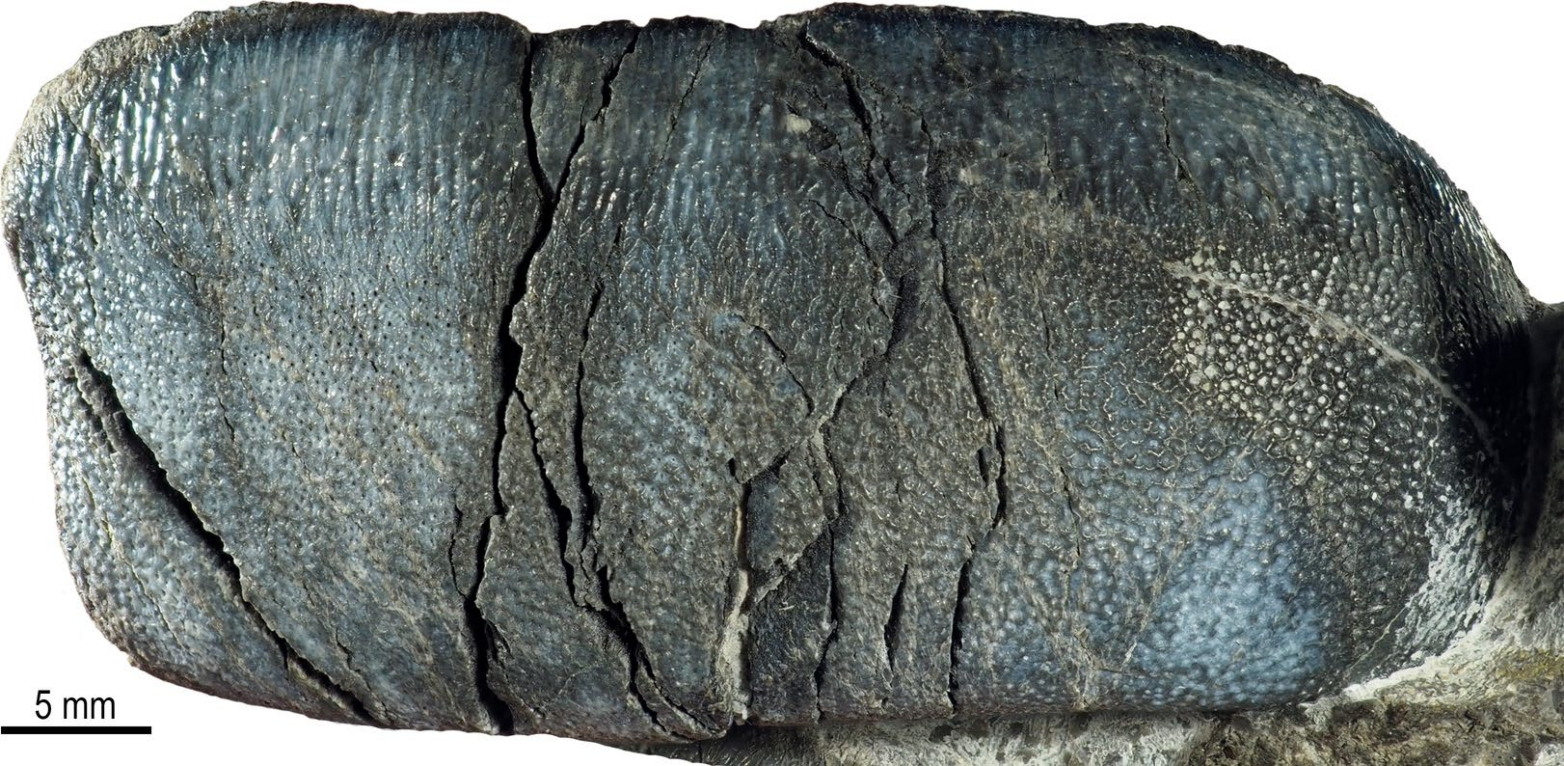
*Strophodus timoluebkei* sp. nov. and details of the crown of the lateral tooth PIMUZ A/I 5724-b in occlusal view

The fossil studied here comprises three complete teeth embedded in a small limestone block (Figs. 2, 3, and 4). The specimen PIMUZ A/I 5724 is deposited at the Department of Paleontology of the University of Zurich, and it was prepared by using a pneumatic airtool device. All photographs were obtained using an Olympus OM-D E-MarkII with a 30 mm Macro Objective.

### Geographical and geological framework

PIMUZ A/I 5724 was surface collected by Mr. Timo Lübke in 2023. The place of discovery is in the talus on the south-eastern slope of the “Chli Mythen”, at an altitude of 1420 masl (47° 2′12.90″N, 8°41′10.42″E), southwest of Brunni, canton Schwyz, Central Switzerland (Fig. 1). The scree consists almost exclusively of loose material from the cliff above. According to Hantke et al. (2022a), the cliff exposes tectonically inverted limestones of the Sulzfluh Limestone Fm. (‘Malmkalk’ according to Weiss, 1949). This formation was documented by Weiss (1949) based on a profile at the neighbouring “Gross Mythen” and placed in the Middle Oxfordian to Late Tithonian based on microfossils observed in thin sections. At Rotenflue, less than 2 km southeast, Boller (1963) was able to date sediments for the youngest part of the Sulzfluh Limestone as Berriasian based on tintinnids. There, however, the stratigraphy is more completely preserved, while on the Gross Mythen the Sulzfluh Limestone Fm. is directly overlain by the Couches Rouges (Albian–Maastrichtian) over a transgression surface, the Sciernes-d’Albeuve Fm. (Berriasian to Barremian) is preserved between the Sulzfluh limestones and Couches Rouges at Rotenflue.

At Gross Mythen, the sequence of the Sulzfluh Limestone Fm. begins with a hiatus above the underlying Griggeli Fm. (Callovian to Early Oxfordian). The lowest 15 m of the Sulzfluh Limestone, which is around 350 m thick (Fig. 1), consists of grey micritic limestone containing radiolarians and pelagic crinoids. The main part of the Sulzfluh Limestone consists of massive, light to medium grey, fine-grained to dense mostly micritic limestone with subordinate brecciated sedimentary layers and mostly resedimented corals (Weiss, 1949). Autochthonous reef limestone is not documented, and the environment is interpreted as neritic to pelagic. The top of the Sulzfluh Limestone at “Gross Mythen” is formed by an approximately one-meter-thick iron-rich calpionellid-bearing limestone. This is followed by the transgressive surface mentioned above and the hemipelagic Couches Rouges Fm. (Albian to Maastrichtian; Hantke et al., 2022a; Weiss, 1949). At Chli Mythen, the transition to the overlying formation (and possibly the uppermost part of the Sulzfluh Limestone Fm.) is not preserved.

Tectonically, Chli Mythen is part of the recumbent limb of the Mythen anticline of the Mythen-Roggenegg thrust and is thus part of the Middle Penninic Klippen nappe (Hantke et al., 2022a). The Mesozoic sediments thus originate from the middle penninic Briançonnais microcontinent. They were sheared off from their bedrock during the Alpine orogeny, folded and thrusted northwards onto younger sediments of the northern continental margin.

Due to the local setting of the discovery and the relatively homogeneous composition of the Sulzfluh Limestone, it is not possible to identify from which stratigraphic subunit within the section PIMUZ A/I 5724 originated. Moreover, the lithology of the rock displays the ‘normal facies’ of the Sulzfluh Limestone Fm. The geological map and stratigraphic chart shown in Fig. 1 is based on data from Weiss (1949), Hantke et al., (2022a, 2022b) and swisstopo (https://map.geo.admin.ch and https://www.swisstopo.admin.ch/en/geological-atlas-of-siwtzerland-1-25000).

### Comparisons and taxonomy

Taxonomic identification is based on an extensive bibliographical review and anatomical comparison with fossil specimens housed in the following Swiss collections: Haimuseum und Sammlung R. Kindlimann’ in Aathal-Seegraeben, JURASSICA Museum (MJSN) in Porrentruy, Natural History Museum of Basel (NMB), and the collection of the Department of Palaeontology of the University of Zurich (PIMUZ). Tooth descriptive terminology follows Cappetta (2012), Rees and Underwood (2008), Rigal and Cuny (2016), and Stumpf et al. (2023). Systematic placement follows Maisey (1989) and Stumpf et al., (2021, 2023).

### Systematic palaeontology

Chondrichthyes Huxley, 1880

†Hybodontiformes Patterson, 1966

†Hybodontidae Owen, 1846

†Acrodontinae Casier, 1959 sensu Maisey, 1989

†*Strophodus* Agassiz, 1838

#### Type species

*Strophodus longidens* Agassiz, 1838

#### Recognized species

*Strophodus atlasensis* Stumpf et al., 2023: Bajocian, Morocco; Bathonian, India.

*Strophodus dunaii* (Szabó & Főzy, 2020): Lower Jurassic (Aalenian), Hungary.

*Strophodus indicus* Sharma & Singh, 2021: Bathonian, India.

*Strophodus jaisalmerensis* Kumar et al., 2022: Bathonian, India.

*Strophodus longidens* Agassiz, 1838 (type species): Bathonian, France.

*Strophodus magnus* Agassiz, 1838: Lower–Middle Jurassic, England, France, India (Bhosale et al., 2024; Rees & Underwood, 2008; Rigal & Cuny, 2016; Sharma & Singh, 2021).

*Strophodus medius* Owen, 1869: Bathonian, France, England, India (Rees & Underwood, 2008; Sharma & Singh, 2021).

*Strophodus rebecae* Carrillo-Briceño & Cadena, 2022: Lower Cretaceous, Colombia.

*Strophodus reticulatus* Agassiz, 1838: Bathonian–Tithonian, England, France, Germany, Hungary, Switzerland (Rieppel, 1981; Stumpf et al., 2022 and references therein).

*Strophodus smithwoodwardi* (Peyer, 1946): Toarcian, Switzerland.

*Strophodus subreticulatus* Agassiz, 1838: Kimmeridgian: Switzerland. *Strophodus tenuis* Agassiz, 1838: Aalenian–Bathonian, Germany, England (Rees & Underwood, 2008).

*Strophodus udulfensis* (Leuzinger et al., 2017): Kimmeridgian, Switzerland, Poland (Stumpf et al., 2022).

*Strophodus timoluebkei* sp. nov.

(Figs. 2, 3, and 4).

*Etymology* In honor of Mr. Timo Lübke, who found the specimen and kindly donated it to the paleontological collection of the University of Zurich.

*Holotype* PIMUZ A/I 5724, three teeth and a fragment of another in light grey limestone matrix.

*Type locality and horizon* South-east flank of the “Chli Mythen”, Sulzfluh Limestone Fm. (Middle Oxfordian to Late Tithonian).

*Referred material* The holotype and only specimen of *Strophodus timoluebkei* sp. nov. is represented by one anterior and two lateral crushing-type teeth likely from the same individual in semi-articulated position. Most of the tooth roots are still embedded or partially exposed in limestone matrix. Only a small fragment of the root of a fourth tooth is preserved and embedded in the same block. The assignment of these teeth to the upper or lower jaw is not possible due to the incompleteness of the material. The ZooBank LSIDs (Life Science Identifiers) for this publication is: urn:lsid:zoobank.org:pub:08973738-4EED-4F8A-B727-FEADD716FA3F.

#### Diagnosis

Hybodontiform shark-like chondrichthyan species with a crushing-type dentition distinguished by the following features: (1) high degree of crown heterodonty between anterolateral and lateral teeth. (2) anterolateral teeth with subtriangular shape, lacking occlusal crest and characterized by a reticular ornamentation with well-defined folds that descend from the domed crown. (3) lateral teeth of the second lateral file with a subrectangular shape and wider mesio-distally than labio-lingually. The crown is high, slightly domed in the mesial part and lacking an occlusal crest, with an ornamentation that consists of a finely pitted reticulated pattern on most labial sections of the crown, which turn into numerous strong parallel folds labio-lingually aligned along the lingual section of the crown. Labial border is vertical and the lingual one inclined and shorter, and the root is high with a flat base.

#### Description

*Anterolateral tooth*. PIMUZ A/I 5724-a is a mesio-distally short tooth (20 mm in length, 12 mm wide and 12 mm high) with a subtriangular shape in occlusal outline and a domed crown that lacks an occlusal crest (Fig. 2). The ornamentation consists of a reticular pattern with well-defined folds that descend from the dome of the crown. The enameloid of the lingual edge is ornamented with irregular vertical ridges, and the labial border and part of the root of this section are missing. The root section corresponding to the lingual surface is embedded in the matrix and thus, it cannot be examined. In general, PIMUZ A/I 5724-a appears to have a high root. The other isolated root fragment embedded in the matrix could belong to an anterior tooth, however, it does not display any diagnostic features.

*Lateral teeth*. Both lateral teeth PIMUZ A/I 5724-b and PIMUZ A/I 5724-c likely belong to the second lateral file. They have well-preserved crowns and are larger than the anterolateral tooth (Fig. 3). The crowns are subrectangular in shape with a weakly lingually arched contour in occlusal outline. They are wider mesio-distally than labio-lingually long (PIMUZ A/I 5724-b: 51 mm length, 24 mm wide, and 20 mm high; PIMUZ A/I 5724-c: 49 mm length, 28 mm wide, and 20 mm high). The mesial part of the crown is thicker than the distal one and it becomes slightly domed, and no crest is visible. The labial border is vertical (90° to the occlusal surface, Fig. 3F), while the lingual one is more inclined and somewhat shorter (Fig. 3G). The enameloid of the labial border is densely ornamented with fine irregular vertical ridges, somewhat reticulated, while on the lingual border, it is finely reticulated and pitted. The occlusal ornamentation consists of a finely pitted reticulated pattern on most labial sections of the crown, which turns into numerous parallel strong folds labio-lingually aligned along the lingual part of the crow (Figs. 3, 4). PIMUZ A/I 5724-c shows what appears to be slight signs of functional wear on the labial section of the crown (Fig. 3C), although we do not rule out that this could also be the result of erosion. Much of the root of both lateral teeth is still embedded in the matrix (e.g., Fig. 2), but the exposed parts show that it is high, with a flat base and it probably does not protrude beyond the lingual and labial edges of the crown. Foramina are randomly distributed all over the root, and small foramina are arranged in a line that follows the base of the crown on the labial side.

### Comparison

Morphological features used for species identification in *Strophodus* focus on dental traits due to the lack of other diagnostic skeletal elements (Stumpf et al., 2022, 2023). Consequently, we compare *Strophodus timoluebkei* sp. nov. from the Upper Jurassic of Switzerland to the other 13 *Strophodus* species from the Jurassic of Africa, Asia and Europe, and Late Cretaceous of South America (Carrillo-Briceño & Cadena, 2022; Kumar et al., 2022; Rees & Underwood, 2008; Sharma & Singh, 2021; Stumpf et al., 2022, 2023; Szabó & Főzy, 2020); the diagnostic differences are presented below.

*Strophodus timoluebkei* sp. nov. differs from *S. atlasensis* from the Lower–Middle Jurassic of Morocco and India (Bhosale et al., 2024; Stumpf et al., 2023), since the latter is characterized by bulbous teeth of the second lateral file with a rather flat crown with a typical subrectangular to bean-shaped occlusal outline and a finely reticulate ornamentation consisting of very small densely packed pits (Stumpf et al., 2023, Figs. 2, 3). *Strophodus atlasensis* was described based on an incomplete articulated well-preserved dentition, in which the anterior teeth are missing, and their morphology is thus unknown.

*Strophodus dunaii* from the Aalenian of Hungary can be differentiated from *S. timoluebkei* sp. nov., by their teeth displaying a low and wide transversal ridge that runs along the entire crown surface and by the complex ornamentation composed of branching folds and ridges at the higher crown parts turning into a minutely reticulate pattern towards the crown edges (Szabó & Főzy, 2020, Figs. 2–3).

*Strophodus indicus*, another species from the Bathonian of India, has mesio-distally elongated, narrower lateral teeth, which are rectangular in shape, with a flat crown with an occlusal ornamentation composed of prominent enameloid folds forming a ridge-and-groove-pattern (Sharma & Singh, 2021, Fig. 4).

*Strophodus timoluebkei* sp. nov., can be differentiated from *S*. *jaisalmerensis* from the Bathonian of India by the latter having mesiodistally elongated lateral teeth with a parallelogram-shaped outline, with a remarkably thin and almost flat or gently sinuous, domed crown bearing a uniformly finely reticulated ornamentation across the entire crown (Kumar et al., 2022, Figs. 4, 5). The anterior teeth *S*. *jaisalmerensis*, although with a sub-triangular to rhomboidal occlusal outline (Kumar et al., 2022, Fig. 4h–i), appear to be more elongated than those from *S. timoluebkei* sp. nov. and lack well-defined folds that descend from the dome of the crown.

**Fig. 5:**
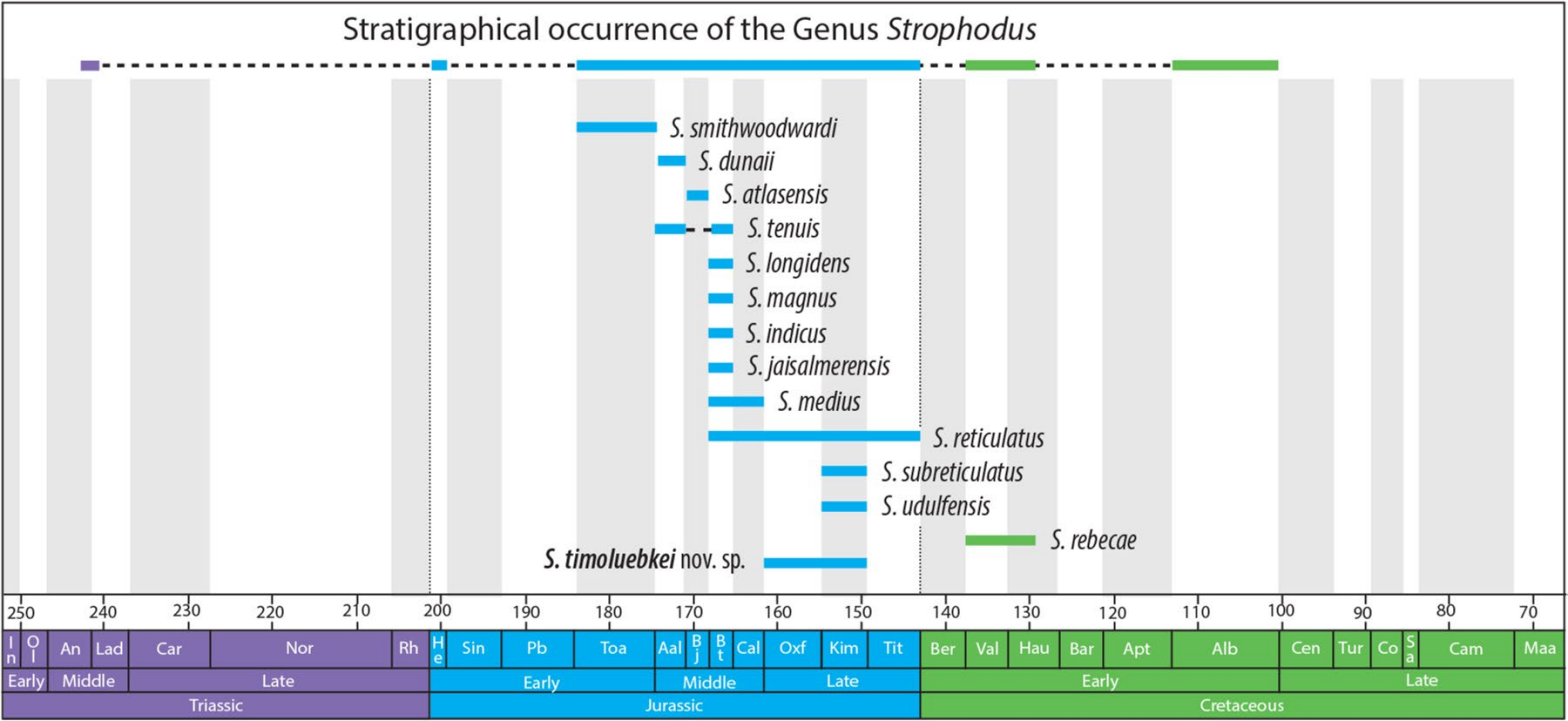
The spatial and temporal distribution of *Strophodus*. Based on Stumpf et al. (2023)

*Strophodus timoluebkei* sp. nov. differs from *S*. *magnus* from the Bathonian of Europe and India in the teeth of the latter species having a second lateral file with a quadrangular, mesiodistally elongated and flat or weakly domed crown with an expanded mesial extremity projected labially and with a uniformly finely reticulate ornamentation (Bhosale et al., 2024; Rees & Underwood, 2008; Rigal & Cuny, 2016; Sharma & Singh, 2021). Anterior teeth in both *S*. *magnus* and *S. timoluebkei* sp. nov. have a subtriangular outline, but these teeth of the former species have an ornamentation, which is uniformly and finely reticulated (Rees & Underwood, 2008; Rigal & Cuny, 2016). In contrast, the anterior teeth of *S. timoluebkei* sp. nov. appear to have a more domed crown with a reticular pattern with well-defined folds that descend from the dome.

*Strophodus medius* from the Bathonian of England, France, and India, was described based on an articulated dentition (Rees & Underwood, 2008, Fig. 3). Its lateral teeth from the second file, defined as fourth row teeth by Rees and Underwood (2008), are characterized by a mesio-distally elongate rectangular outline with a reticulate ornamentation completely covering the occlusal part of the teeth. These teeth are more elongated than those of *S. timoluebkei* sp. nov. The anterior teeth of *S*. *medius* have crowns highly domed with prominent occlusal crests, characters not observed in our specimen PIMUZ A/I 5724-a.

In comparison to *Strophodus rebecae* from the Valanginian–Hauterivian of Colombia, this species has relatively short lateral teeth with a characteristic parallelogram outline shape with a pointed and lingually oriented mesial extremity and with a less domed crown with a finely pitted reticulated pattern (Carrillo-Briceño & Cadena, 2022, Fig. 2). The shorter lateral teeth in *S*. *rebecae* differentiate this species from *S. timoluebkei* sp. nov. and other Jurassic *Strophodus* species.

*Strophodus timoluebkei* sp. nov. anterolateral teeth differ from those of *S*. *reticulatus* from the Middle Triassic (*S*. cf. *reticulatus* according to Rieppel, 1981) and Middle Jurassic of Europe in possessing crowns lacking an occlusal crest with a strong ornamentation characterized by radiating ridges (Agassiz, 1838, plate 17; Stumpf et al., 2021, Figs. 7S–7U; Brignon, 2023, Fig. 10). Anterolateral teeth of *S*. *reticulatus* are characterized by having strongly domed crowns with occlusal crests (Brignon, 2023, Fig. 10, 11). In reference to the *S*. *reticulatus* teeth from the Callovian of the Oxford Clay at Fletton (UK), these, according to Brignon (2023), appear to belong to a different species of *Strophodus*. Originally, these teeth were referred to “*Asteracanthus ornatissimus* var. *flettonensis*” by Woodward (1888, 1889), and later to *A. ornatissimus* or *S. reticulatus* by other authors (see Brignon, 2023, pp. 19–20). Brignon (2023) designated the teeth from the Callovian of the Oxford Clay under the new combination *Strophodus flettonensis* (Woodward, 1888), arguing that lateral teeth are characterized by a slight occlusal ridge parallel to the labial and lingual borders, while the lateral teeth of *S*. *reticulatus* lack this ridge and are relatively wider in their labiolingual direction, giving them a more robust appearance. The anterolateral and lateral teeth of the Oxford Clay differ also from the teeth of *S. timoluebkei* sp. nov. (see references in Brignon, 2023, pp. 19–20), and a further redescription of these Oxford Clay specimens is needed to validate them as a species distinct from *S*. *reticulatus*.

*Strophodus subreticulatus* from the Kimmeridgian of Switzerland differs from *S. timoluebkei* sp. nov. in having lateral teeth that appear to be wider mesio-distally, attaining a parallelogram-shaped occlusal outline with flat to slightly domed crowns (Agassiz, 1838, plate 18, Figs. 5–10).

*Strophodus timoluebkei* sp. nov. differs from *S. smithwoodwardi* from the Toarcian of Switzerland in the presence of lateral teeth in the second file with a more rectangular outline, with a straighter labial edge, a thicker and slightly domed crown in the mesial section, and an ornamentation with finely pitted reticulated pattern and numerous parallel strong folds labio-lingually aligned along the lingual part of the crow. In contrast, the equivalent teeth of the holotype of *S*. *smithwoodwardi* (see Peyer, 1946, plate 2) have less rectangular outlines with the labial and lingual edges more concave and convex, respectively, and the crown is less domed in the mesial section with a finer reticular ornamentation lacking a “pitted pattern” as in teeth PIMUZ A/I 5724b–c. The most significant difference between the lateral teeth of these two species is that in *S*. *smithwoodwardi* the lingual edge of the crown tends to be angled (acute angle) in profile view with a characteristic smooth surface (Peyer, 1946, plate 2, Fig. 7), while in *S. timoluebkei* sp. nov., the lingual edge is shorter, slightly inclined, and the enameloid is densely covered with fine irregular vertical ridges (Fig. 3G). The smooth and angled lingual edge is a feature that we have observed in all the *S*. *smithwoodwardi* lateral teeth described by Peyer (1946). The anterior teeth in the new species described here and those of *S*. *smithwoodwardi* (Peyer, 1946, plate 4) are similar in occlusal outline, but the crown is more domed and heavily ornamented in *S. timoluebkei* sp. nov.

*Strophodus tenuis* from Aalenian–Bathonian strata of England and Germany is differentiated from *S. timoluebkei* sp. nov. mainly by the presence, in the former, of mesio-distally more elongate and slenderer anterolateral and lateral teeth with a sigmoid curvature in occlusal outline and well developed and asymmetrically situated domed areas (see Agassiz, 1838, plate 18, Figs. 16–25; Rees & Underwood, 2008, plate 5, Figs. 12–16). The ornamentation of lateral teeth of *S. tenuis* is characterized by folds that form a reticulate pattern with smooth areas on the lateral sides (Rees & Underwood, 2008). *Strophodus longidens* from the Bathonian of northern France, which is the type species of the genus, can be differentiated from *S. timoluebkei* sp. nov. mainly by the many mesio-distally elongate anterolateral and lateral teeth, especially the last ones having asymmetrically situated domed areas (Agassiz, 1838, plate 16, Figs. 1–8). No detailed information on tooth ornamentation was provided by Agassiz (1838), and apparently, the holotype was destroyed during the Second World War (Rees & Underwood, 2008; Szabó & Főzy, 2020).

The second lateral teeth of *S*. *udulfensis* from the Kimmeridgian of Switzerland and Poland are mesio-distally elongated with a parallelogram-shaped occlusal outline, a domed crown in the mesial part with a strong reticulated ornamentation that is smaller and more densely packed along the outline of the crown (Leuzinger et al., 2017, Fig. 7). The morphological pattern of the anterior and lateral teeth of *S*. *udulfensis* allows a differentiation from *S. timoluebkei* sp. nov.

## Discussions and concluding remarks

The hybodontiform shark-like *Strophodus* has a stratigraphic range of about 130 million years (Fig. 5), spanning from the Middle Triassic to the Early Cretaceous age (Stumpf et al., 2023, Fig. 4). Its geographic distribution was also wide with a fossil record that ranges from Africa, Asia, and Europe to South America (Cappetta, 2012; Stumpf et al., 2023, and references therein). Given the lack of skeletal material, morphological characters for species assignment within the genus focus primarily on its dental features. *Strophodus* is characterized by its highly specialized crushing-type dentition with a high degree of crown heterodonty (Cappetta, 2012), which is divided into symphyseal (only for the lower jaw; see Pfeil, 2011), anterior, lateral and posterior teeth (Rigal & Cuny, 2016). Of the previously described thirteen *Strophodus* species currently accepted as valid (Bhosale et al., 2024), only a few (Stumpf et al., 2023, tab. 1) are known by articulated dentitions or associated teeth. *Strophodus timoluebkei* sp. nov. is described here based on associated dental elements of which we know, for now, only the morphology of the anterolateral and lateral teeth, whose differences in crown shape suggests a more pronounced heterodonty than in the other species of the genus. The differential dental diagnosis presented above for the anterolateral and lateral teeth of the new species from the Sulzfluh Limestone allow us to separate it based on tooth morphology from the other 13 *Strophodus* species recognized from the Jurassic of Africa, Asia and Europe (Agassiz, 1838; Kumar et al., 2022; Peyer, 1946; Rees & Underwood, 2008; Sharma & Singh, 2021; Stumpf et al., 2023; Szabó & Főzy, 2020, and references therein) and the Lower Cretaceous of South America (Carrillo-Briceño & Cadena, 2022).

The new information presented here sheds light on the paleodiversity of *Strophodus*, increasing the number of known species to 14. However, this number of species is underestimated, since there are several specimens deposited in European collections that correspond to species not yet described. Some examples include (1) the most complete and associated set of jaws with teeth known from the Solnhofen Archipelago (Tithonian of Germany) and described by Pfeil (2011) as *Asteracanthus* sp., and (2) a specimen from the Monte San Giorgio Lagerstätte, Middle Triassic of Switzerland (which is the oldest *Strophodus* known) and reported by Rieppel (1981) as *S*. cf. *reticulatus*, but it seems to be different from this taxon and other species of *Strophodus* by possessing a higher number of anterior tooth files (S. Stumpf, pers. comm., 2022), so a re-examination of the material is needed to determine its precise systematic position. According to Stumpf et al. (2023), the western Tethys region may have been the center of geographic origin of *Strophodus* considering the oldest record of the genus from the Middle Triassic of the Monte San Giorgio Lagerstätte (Rieppel, 1981). The fossil record suggests that *Strophodus* had its peak diversity during the Middle and Late Jurassic (Fig. 5), with a diversity fluctuation that significantly dropped by the Early Cretaceous (Stumpf et al., 2023), the demise likely having several causes, perhaps involving both changes in availability of preferred food resources (Tennant et al., 2017) and an increasing competitive niche overlap with other groups of durophagous elasmobranchs, which rapidly diversified during the Jurassic and Cretaceous (Guinot & Cavin, 2016).

To our knowledge, *Strophodus timoluebkei* sp. nov., represents the only record of vertebrates so far known from the Sulzfluh Limestone Fm. of the Klippen nappe. Apart from coral fragments, only very few other macro-fossils from the monotonous Sulzfluh Limestone sequence of Gross Mythen and Chli Mythen are described in the literature (fragments of bivalves, ammonites, pelagic crinoids; Smit Sibinga, 1921). They do not allow for stratigraphic subdivision, though.

The Jurassic to Cretaceous sediments of the Klippen nappe were deposited on a microcontinent in the Penninic Ocean; the Piedmont-Ligurian Ocean was situated to the south and north of the microcontinent, the narrow Valais Trough opened up. Within this microcontinent, a shallower southern zone and a deeper, more pelagic northern zone can be distinguished. According to Weiss (1949), the sediments of the Gross Mythen and Chli Mythen may have been deposited between the neritic and pelagic zones. The resedimented corals and the frequent breccias as well as the absence of actual reefs as limestone support this assessment.

The assignment of a neritic to pelagic environment of this geological unit (Hantke et al., 2022a), suggests that *Strophodus timoluebkei* sp. nov. was adapted to a marine ecosystem, as it was also inferred for other *Strophodus* species (Carrillo-Briceño & Cadena, 2022; Kumar et al., 2022; Rees & Underwood, 2008; Stumpf et al., 2023; Szabó & Főzy, 2020, and references therein) although an estuarine environment (Bhosale et al., 2024) and other freshwater-influenced environments (Leuzinger et al., 2015) have been suggested for *Strophodus*.

## Data Availability

No datasets were generated or analysed during the current study.

## References

[CR1] Agassiz, L. J. R. (1837). *Recherches sur les poissons fossiles* (Vol. 3). Imprimerie de Petitpierre.

[CR2] Agassiz, L. J. R. (1838). *Recherches sur les Poissons fossiles* (Vol. 3). Imprimerie de Petitpierre.

[CR3] Bhosale, S., Rakshit, N., Natarajan, A., Chauhan, G., & Thakkar, M. (2024). The oldest Gondwanan record of durophagous shark *Strophodus* Agassiz, 1838 (Hybodontiformes) from the Kachchh Basin, western India. *Historical Biology,**37*(3), 650–662. 10.1080/08912963.2024.2324430

[CR4] Boller, K. (1963). Stratigraphische und mikropaläontologische Untersuchungen im Neocom der Klippen-Decke (östlich der Rhone). *Eclogae Geologicae Helvetiae,**56*(1), 15–102.

[CR5] Brignon, A. (2023). Révision des vertébrés du Calcaire de Tonnerre (Jurassique supérieur, Yonne) au travers des collections du XIXe siècle et en particulier de celle de Charles Rathier (1812–1888). *Bulletin D’information des Géologues Du Bassin De Paris,**60*(1), 3–36.

[CR6] Carrillo-Briceño, J. D., & Cadena, E.-A. (2022). A new hybodontiform shark (*Strophodus* Agassiz, 1838) from the Lower Cretaceous (Valanginian-Hauterivian) of Colombia. *PeerJ,**10*, Article e13496. 10.7717/peerj.13496 35673391 10.7717/peerj.13496 PMC9167585

[CR7] Casier, E. (1959). Contributions à l’étude des Poissons fossils de la Belgique, XII –Sélaciens et Holocéphales sinémuriens de la Province de Luxembourg. *Bulletin De L’institut Royal des Sciences Naturelles De Belgique,**38*(8), 1–35.

[CR8] Guinot, G., & Cavin, L. (2016). ‘Fish’ (Actinopterygii and Elasmobranchii) diversification patterns through deep time. *Biological Reviews,**91*, 950–981. 10.1111/brv.12203 26105527 10.1111/brv.12203

[CR9] Hantke, R., Trümpy, R., Baumeler, A., Bollinger, D., Felber, P. Letsch, D. & Grünig, A. 2022b. Blatt 1152 Ibergeregg.—Geol. Atlas Schweiz 1:25000, Karte. 175.

[CR10] Hantke, R., Letsch, D., Felber, P., Baumeler, A., Heinz, R., Uttinger, J., & Grünig, A. 2022a. Blatt 1152 Ibergeregg.—Geol. Atlas Schweiz 1:25000, Erläut. 175. (in German)

[CR11] Huxley, T. H. (1880). On the application of the laws of evolution to the arrangement of the Vertebrata, and more particularly of the Mammalia. *Proceedings of the Zoological Society of London,**43*, 649–662.

[CR12] Kumar, K., Bajpai, S., Pandey, P., Ghosh, T., & Bhattacharya, D. (2022). Hybodont sharks from the Jurassic of Jaisalmer, Western India. *Historical Biology,**34*(6), 953–963. 10.1080/08912963.2021.1954920

[CR13] Leuzinger, L., Cuny, G., Popov, E., Billon-Bruyat, J. P., & Johanson, Z. (2017). A new chondrichthyan fauna from the Late Jurassic of the Swiss Jura (Kimmeridgian) dominated by hybodonts, chimaeroids and guitarfishes. *Papers in Palaeontology,**3*(4), 471–511. 10.1002/spp2.1085

[CR14] Leuzinger, L., Kocsis, L., Billon-Bruyat, J. P., Spezzaferri, S., & Vennemann, T. (2015). Stable isotope study of a new chondrichthyan fauna (Kimmeridgian, Porrentruy, Swiss Jura): An unusual freshwater-influenced isotopic composition for the hybodont shark *Asteracanthus*. *Biogeosciences,**12*(23), 6945–6954. 10.5194/bg-12-6945-2015

[CR15] Maisey, J. G. (1987). Cranial anatomy of the lower Jurassic shark *Hybodus reticulatus* (Chondrichthyes: Elasmobranchii), with comments on hybodontid systematics. *American Museum Novitates,**2878*, 1–39.

[CR16] Maisey, J. G. (1989). *Hamiltonichthys mapesi*, g. & sp. nov. (Chondrichthyes; Elasmobranchii), from the Upper Pennsylvanian of Kansas. *American Museum Novitates,**2931*, 1–42.

[CR17] Owen, R. (1846). *Lectures on the comparative anatomy and physiology of the vertebrate animals, delivered at the Royal College of Surgeons of England in 1844 and 1846, Part 1*. Longman.

[CR18] Patterson, N. C. (1966). British Wealden sharks Bulletin of the British Museum (Natural History). *Geology,**11*, 283–350.

[CR19] Peyer, B. (1946). Die schweizerischen Funde von Asteracanthus (Strophodus). *Schweizerische Palaeontologische Abhandlungen,**64*, 1–103.

[CR20] Pfeil, F. H. (2011). Ein neues Asteracanthus-Gebiss aus den Kieselplattenkalken (Oberjura, Tithonium, Malm Zeta 3, Mörnsheim-Formation) des Besuchersteinbruchs in Mühlheim. *Jahresbericht 2010 und Mitteilungen der Freunde der Bayerischen Staatssammlung Für Paläontologie und Historische Geologie München,**39*, 36–60.

[CR21] Rees, J., & Underwood, C. J. (2008). Hybodont sharks of the English Bathonian and Callovian (middle Jurassic). *Palaeontology,**51*(1), 117–147. 10.1111/j.1475-4983.2007.00737.x

[CR22] Rieppel, O. (1981). The hybodontiform sharks from the Middle Triassic of Mte, San Giorgio, Switzerland. *Neues Jahrbuch Für Geologie und Paläontologie, Abhandlungen,**161*, 324–353.

[CR23] Rigal, S., & Cuny, G. (2016). On the rarity of anterior teeth of *Asteracanthus magnus* (Euselachii: Hybodontiformes). *Neues Jahrbuch Für Geologie und Paläontologie,**279*(1), 35–41.

[CR24] Sharma, A., & Singh, S. (2021). A small assemblage of marine hybodont sharks from the Bathonian of the Jaisalmer Basin, India. *Neues Jahrbuch Für Geologie und Paläontologie,**301*(3), 317–333. 10.1127/njgpa/2021/1014

[CR25] Smit Sibinga, G. L. (1921). *Die Klippen der Mythen und Rotenfluh*. Hannover: Gebrüder Jänecke.

[CR26] Stumpf, S., Kettler, C., Kindlimann, R., Cuny, G., & Kriwet, J. (2023). The oldest Gondwanan record of the extinct durophagous hybodontiform chondrichthyan, *Strophodus* from the Bajocian of Morocco. *Swiss Journal of Palaeontol,**142*, 5. 10.1186/s13358-023-00270-w

[CR27] Stumpf, S., López-Romero, F. A., Kindlimann, R., Lacombat, F., Pohl, B., & Kriwet, J. (2021). A unique hybodontiform skeleton provides novel insights into Mesozoic chondrichthyan life. *Papers in Palaeontology,**7*(3), 1479–1505. 10.1002/spp2.1350

[CR28] Stumpf, S., Meng, S., & Kriwet, J. (2022). Diversity Patterns of Late Jurassic Chondrichthyans: New insights from a historically collected hybodontiform tooth assemblage from Poland. *Diversity,**2022*(14), 85. 10.3390/d14020085

[CR29] Szabó, M., & Főzy, I. (2020). Asteracanthus (Hybodontiformes: Acrodontidae) remains from the Jurassic of Hungary, with the description of a new species and with remarks on the taxonomy and paleobiology of the genus. *Neues Jahrbuch Für Geologie und Paläontologie. Abhandlung,**297*(3), 295–309. 10.1127/njgpa/2020/0926

[CR30] Tennant, J. P., Mannion, P. D., Upchurch, P., Sutton, M. D., & Price, G. D. (2017). Biotic and environmental dynamics through the Late Jurassic-Early Cretaceous transition: Evidence for protracted faunal and ecological turnover. *Biological Reviews,**92*(2), 776–814. 10.1111/brv.12255 26888552 10.1111/brv.12255 PMC6849608

[CR31] Weiss, H. (1949). *Stratigraphie und Mikrofauna des Klippenmalm*. Technischen Hochschule und der Universität Zürich.

[CR32] Woodward, A. S. (1888). On some remains of the Extinct Selachian *Asteracanthus* from the Oxford Clay of Peterborough, preserved in the collection of Alfred, N. Leeds, Esq., of Eyebury. *Journal of Natural History,**2*, 336–342. 10.1080/00222938809460935

[CR33] Woodward, A. S. (1889). *Catalogue of the Fossil Fishes in the British Museum (Natural History), Part I*. British Museum.

